# Frontoparietal control-default mode connectivity predicts TMS effects on cognitive control

**DOI:** 10.1162/IMAG.a.1298

**Published:** 2026-07-14

**Authors:** Brian Kim, John D. Medaglia

**Affiliations:** Department of Applied Cognitive and Brain Sciences, Drexel University, Philadelphia, PA, United States; Department of Neurology, University of Pennsylvania, Philadelphia, PA, United States

**Keywords:** cognitive control, neuromodulation, TMS, LFPN/MFPN connectivity, DDM, individual differences, latent factor analysis

## Abstract

Transcranial Magnetic Stimulation (TMS) is a promising tool to probe and enhance cognitive control, yet effects are often inconsistent across individuals. These inconsistencies may arise from individual differences in Lateral Frontoparietal (Control) Network (L-FPN) and Medial Frontoparietal (Default) Network (M-FPN) interactions, essential for suppressing internal distraction and facilitating cognitive control. We tested whether baseline connectivity between the L-FPN and M-FPN moderates TMS outcomes in cognitive control tasks. We used the generalized drift rate as a task-general behavioral index of cognitive control, as it overcomes the reliability and interpretability limitations of standard difference measures. Participants completed inhibition (Stroop), working memory (n-back), and flexibility (Navon) tasks before and after intermittent theta-burst stimulation (iTBS) to the L-FPN, dorsal frontoparietal (attention) network (D-FPN), or cranial vertex. Stimulation targets were defined using individualized resting-state parcellations to maximize precision. We found that baseline connectivity moderated stimulation outcomes: individuals with more integrated L-FPN/M-FPN networks benefited most from L-FPN stimulation, whereas those with more segregated networks showed greater improvements from D-FPN stimulation. Notably, stimulation of a single site did not uniformly enhance performance, underscoring the importance of individual network profiles. These findings highlight L-FPN/M-FPN interactions as a basis for individualized TMS interventions and the utility of combining precise network mapping with robust behavioral modeling to optimize neuromodulation interventions.

## 1 Introduction

Researchers have long sought to improve cognitive control, the ability to coordinate mental processes and action with goals (Menon & D’Esposito, 2022). Cognitive control is essential for health (Miller et al., 2011), linked to resilience against neuropathology (Grant et al., 2014; Medaglia et al., 2017), and is impaired in many mental disorders (Grahek et al., 2019; Laurenson et al., 2015). However, TMS often yields inconsistent effects on cognitive control (Dengler et al., 2024; Hernández-Sauret et al., 2024), potentially due to the lack of reliable task-general behavioral and neural measures. Most behavioral tasks isolate distinct subcomponents of cognitive control, such as flexibility, working memory, or inhibition. Accordingly, behavioral outcomes correlate weakly across tasks (Hedge et al., 2022). Although task-general neural markers of control have been identified, they map onto individuals in a highly individual manner (Dengler et al., 2024; Gratton et al., 2018), also limiting their generalizability. Without robust task-general measures, it is difficult to establish benchmarks for theory and to design effective interventions.

One reliable neural marker of cognitive control is the anticorrelation between the lateral frontoparietal network (L-FPN) and the medial frontoparietal network (M-FPN) (Fox et al., 2005). Although these networks are sometimes referred to by their functions as the frontoparietal control network and default mode network respectively, we followed the taxonomy proposed by Uddin et al. (2019), prioritizing anatomical descriptors to facilitate cross-study integration. The L-FPN is a large-scale brain network that activates in response to cognitive control demands (Marek & Dosenbach, 2018), while the M-FPN activates in response to internally-directed or self-generated thought (Andrews-Hanna et al., 2014). Accordingly, L-FPN activation is often dynamically opposed to M-FPN activation, thought to suppress distracting internal thoughts during task performance (Mills et al., 2018). As individuals cannot rely on external cues to regulate internal distraction, successful suppression of the M-FPN may better reflect an individual’s inherent cognitive control ability (Pereira et al., 2020). Supporting this view, stronger L-FPN/M-FPN anticorrelations are associated with greater working memory capacity (Keller et al., 2015), greater control demands (Douw et al., 2016), and developmental improvements (DeSerisy et al., 2021). These findings suggest that L-FPN/M-FPN anticorrelations are a reliable indicator of cognitive control.

Likewise, behavioral measures of task-general cognitive control are often unreliable and hard to interpret. Standard outcomes, such as response time and accuracy, are confounded by speed-accuracy tradeoffs and low reliability of difference scores (Enkavi et al., 2019; Von Bastian et al., 2020). To overcome these limitations, researchers have increasingly used computational models that infer latent cognitive processes from behavioral data. Evidence accumulation models (EAMs; Boag et al., 2025; Ratcliff & McKoon, 2008) view decision-making as a stochastic process in which evidence is gradually accumulated until a decision threshold is reached. A key latent variable in these models, the drift rate, reflects the efficiency of evidence accumulation that jointly incorporates response-time distributions and accuracy. Because sensory input was held constant for each task condition in the current study, individual differences in drift rate specifically reflect cognitive processing efficiency rather than external sensory factors. Thus, EAMs may provide a more interpretable and reliable measure of cognitive control as they mathematically separate processing efficiency (drift rate) from response caution (threshold), both of which are central to the speed-accuracy tradeoff. This distinction is important for cognitive control research, as the drift rate can index the efficiency of goal-directed evidence accumulation, independent of the participant’s chosen strategy regarding caution (threshold). Following standard EAM assumptions (Boag et al., 2025; Ratcliff & McKoon, 2008), we assume within-trial stationarity, where model parameters remain constant for the duration of a single trial, allowing the drift rate to act as an metric for the efficiency of goal-directed processing. By isolating processing efficiency from strategic shifts in response caution, the drift rate may provide a more reliable proxy for cognitive control than traditional difference scores (Enkavi et al., 2019; Weigard et al., 2021). Empirically, higher drift rates predict better performance on control tasks (Alameda et al., 2024) and are associated with activation in L-FPN regions (Gupta et al., 2022). Crucially, joint-modeling approaches simultaneously utilizing behavioral and neural data identified that lower drift rates are associated with M-FPN activation, reflecting less efficient evidence accumulation (Turner et al., 2015). Recent latent-variable approaches have combined drift rates across tasks to derive a generalized drift rate, increasing construct validity by minimizing task-specific noise (Eisenberg et al., 2019; Friedman & Miyake, 2017). Given its psychometric strengths, conceptual grounding, and neural correlates, the generalized drift rate may provide a sensitive and robust behavioral marker that, while not a pure metric, effectively proxies the shared cognitive control mechanisms demanded by these tasks.

In this study, we examined how L-FPN/M-FPN anti-correlation affected TMS responses in cognitive control, measured as changes in the generalized drift rate due to stimulation. This approach also allowed us to test whether the drift rate is a valid metric of performance in cognitive control tasks, and is supported by the L-FPN/M-FPN anti-correlations often related to mind wandering. Participants completed three different externally oriented tasks targeting the core cognitive control functions (Miyake et al., 2000) of inhibition (Stroop), working memory (n-back), and cognitive flexibility (Navon), before and after personalized intermittent theta-burst stimulation (iTBS), an excitatory and efficient form of repeated TMS (Cárdenas-Morales et al., 2010). We used factor analysis to compute generalized drift rate across tasks and linear mixed-effects models to test whether resting-state L-FPN/M-FPN connectivity predicted TMS-induced improvements. We hypothesized that stimulating the L-FPN would increase generalized drift, with the largest gains in individuals with less segregated L-FPN/M-FPN networks, who were expected to show lower baseline performance.

## 2 Methods

### 2.1 Participants

We recruited 21 healthy right-handed participants (10 females, 9 male, 1 “non-binary”, 1 “other”), with an average age of 30.8 (SD = 9.2). Participants were excluded if they had a history of neurological or psychiatric illness.

### 2.2 Ethics Statement

All participants provided written informed consent before data collection occurred. The study protocol was approved by the Institutional Review Board / Human Subjects Committee of the University of Pennsylvania and Drexel University.

### 2.3 Study design

**Fig. 1.**
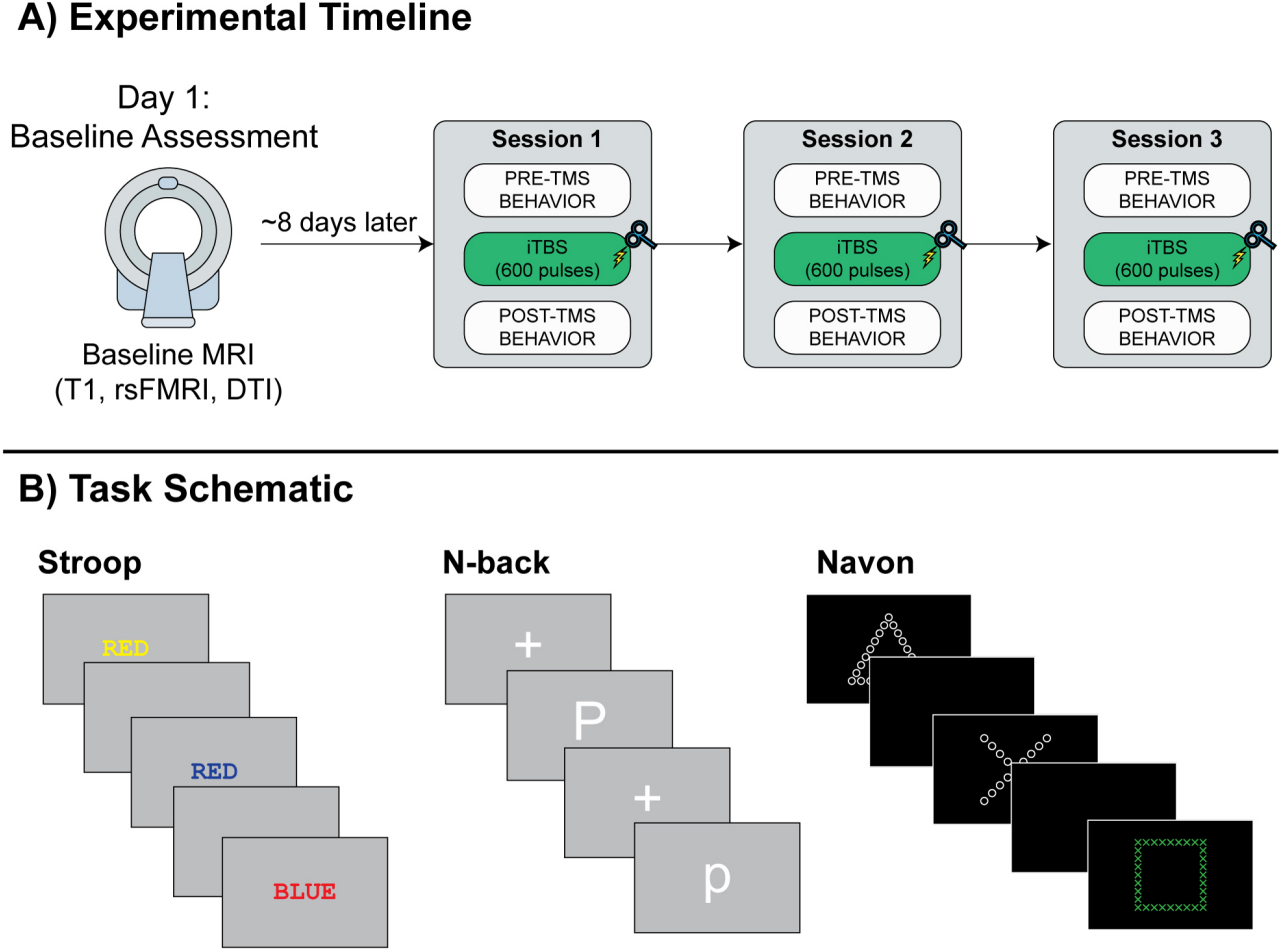
Experimental Timeline and Task Schematics. (A) Experimental Timeline: Participants first underwent a baseline MRI session consisting of structural (T1), resting-state (rsfMRI), and diffusion (DTI) imaging to define individualized network targets. Following baseline, participants completed three iTBS sessions (L-FPN-B, D-FPN, or Vertex) separated by an average of 8.0 days. The order of stimulation sites was counterbalanced across participants to control for potential order and practice effects. Each session included pre-stimulation behavioral task administration, 600 pulses of iTBS delivered to the individualized target, and then post-stimulation behavioral task administration. (B) Task Schematic: Participants performed three cognitive control tasks: 1) the Stroop task (identifying ink color under congruent/incongruent conditions), 2) the N-back task (identifying matches from 0, 1, or 2 trials previous), and 3) the Navon task (switching between global and local features based on color cues).

Each participant completed one baseline MRI session followed by three stimulation sessions (Fig. 1). The MRI session included structural and resting-state scans used to compute individualized network targets (Li et al., 2019; Wang et al., 2015). Each stimulation session consisted of measurements of resting motor threshold, pre-stimulation cognitive control tasks, application of intermittent theta-burst stimulation, and post-stimulation task administration. Stimulation sessions differed only by stimulation site: the lateral frontoparietal network (L-FPN), a passive control site at the cranial vertex, and an active control site in the dorsal frontoparietal (attention) network (D-FPN). The cranial vertex was selected to account for the non-specific physical and neural effects of TMS, such as acoustic clicks, scalp sensations, and general response to TMS. Although vertex stimulation can induce M-FPN deactivations by capturing attention, it does not disrupt the functional connectivity associated with the M-FPN (Jung et al., 2016). The D-FPN was selected as an active control site because it is also strongly activated during externally oriented tasks (Petersen & Posner, 2012; Ptak, 2012), shows behaviorally relevant anticorrelations with the default mode network (M-FPN) (Chai et al., 2012; Spreng et al., 2016), but is more associated with the voluntary orientation of attention (Vossel et al., 2014). By comparing L-FPN stimulation to D-FPN stimulation, we could more effectively isolate the role of the L-FPN independent of generalized attentional effects.

The order of these sessions was fully counterbalanced using a Latin square across participants to reduce potential practice or order effects. Participants without an L-FPN stimulation session were excluded, leaving a final sample of 19 participants. Sessions occurring more than 2.5 standard deviations beyond the mean number of days since baseline were excluded as being excessively delayed. The remaining sessions were separated by an average of 8.02 days (SD = 7.03). Full procedural details can be found in Dengler et al. (2024).

### 2.4 Cognitive control tasks

To address core domains of cognitive control (Miyake et al., 2000), participants performed the following three tasks: 1) the Stroop task for inhibition, where participants identified ink color for congruent, incongruent, and neutral words; 2) the n-back task for working memory, where participants judged whether a stimulus matched a stimulus presented *n* trials earlier (*n* = 0, 1, 2); and 3) the Navon task where participants switched between identifying global and local features of a compound stimulus depending on color cues.

Each task included high- and low-demand conditions. Stimuli, timing, trial counts, and practice procedures followed standard implementations, with counterbalancing of tasks and demands across participants. Full task specifications are provided in the Supplementary Methods.

### 2.5 MRI acquisition and preprocessing

MRI data were acquired on a Siemens 3.0 Tesla Tim Trio whole-body scanner. High-resolution T1-weighted structural and 30-minute resting-state functional images were collected.

Preprocessing used fMRIPrep 21.0.0 (Esteban et al., 2019) with standard corrections for motion, susceptibility, and alignment, followed by nuisance regression using the XCP pipeline (Ciric et al., 2018). Specifically, we implemented a 36-parameter confound regression model that included global signal regression (GSR) to mitigate in-scanner head motion (Satterthwaite et al., 2013). Post-hoc analyses confirmed that our primary moderation findings remained significant when using connectivity measures derived without GSR (see Supplementary Materials).

Individual functional parcellations were derived using the iterative method of Li et al. (2019), which optimizes network boundaries for each participant based on resting-state connectivity. The parcellation returned a pairwise ROI-by-ROI matrix per individual containing Fisher z-transformed correlation coefficients, indicating the functional connectivity between each region pair (Biswal et al., 1995; Li et al., 2019; M. J. Lowe et al., 2000). We defined the between-network connectivity value as the mean of the signed Fisher z-transformed correlation coefficients across all region pairs between the L-FPN and M-FPN. We retained the signs of these coefficients to capture the hypothesized anticorrelation between these networks. Individual node pairs between the networks showed consistent anticorrelations, with 67% of all evaluated connections being negative and 81% of participants showing predominantly negative node-to-node connections between these networks. Full preprocessing steps are also available in the Supplementary Methods.

Recent studies have indicated that the L-FPN is heterogeneous and can be broken into two distinct subsystems with different roles, the L-FPN-A and the L-FPN-B (Dixon et al., 2018). We focused analyses on the L-FPN-B subsystem, which couples with attentional networks and regulates perceptual attention. Likewise, the M-FPN consists of multiple interacting subsystems: a dorsal medial subsystem, a medial temporal subsystem, and a core hub region (Andrews-Hanna et al., 2014). We focused analyses on the M-FPN core subsystem, which consistently anticorrelates with task-positive networks.

### 2.6 TMS targeting

**Fig. 2.**
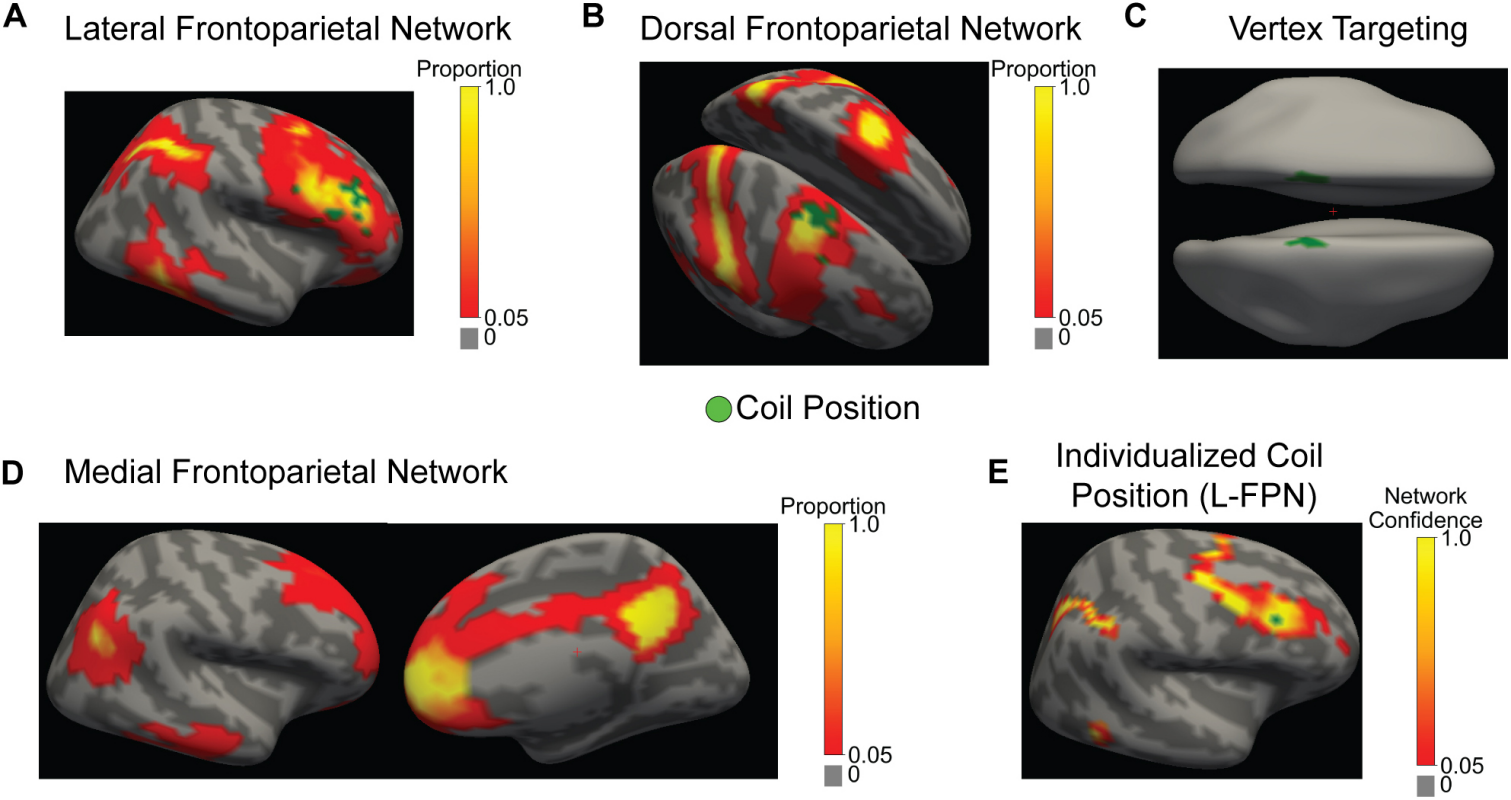
Group-level Probabilistic Network Maps and Targets. Group-level visualization of coil target positions overlaid on the average L-FPN map (A), and average D-FPN map (B). Each green dot represents the coil position of a single participant. The color scale shows the proportion of the sample with network membership in that region, highlighting the inter-subject variability of these network mappings. Cranial vertex maps are shown in (C). Aggregated default mode network maps are shown in (D). Individual-level targeting validation is shown in (E). The heatmap shows the network confidence values for an example subject and chosen target for the L-FPN. The target marker is within the high-confidence region of the L-FPN near the DLPFC, showing the ability of our procedure to account for individual anatomical variance.

Stimulation targets were identified from individualized network maps, selecting peak-confidence voxels within the L-FPN-B or D-FPN, while avoiding adjacent or overlapping regions. For the L-FPN-B, targets were located in the middle frontal gyrus, excluding areas with L-FPN-A affiliation to ensure subsystem specificity. For the D-FPN, we targeted a region anterior to the precentral gyrus near the frontal eye fields. The vertex target was defined as the point nearest the central sulcus between the pre- and postcentral gyri. Average MNI coordinates are provided in Table 1 and displayed in Figure 2.

**Table 1.** Mean stimulation target coordinates and variability.

Target region	Mean coordinate [x, y, z]	Standard deviation [x, y, z]
L-FPN-B	[48.6, 26.7, 23.3]	[4.8, 8.3, 6.2]
D-FPN	[24.1, -2.3, 62.8]	[3.0, 4.5, 2.7]
Vertex	[-0.6, -33.2, 70.8]	[1.3, 5.3, 3.9]

Each value represents coordinates in standard MNI space. The L-FPN-B has more spread, aligning with prior studies indicating higher variability in these regions (Gratton et al., 2018). By contrast, the D-FPN and Vertex targets exhibit higher consistency, with the Vertex showing minimal lateral deviation (x- direction) due to midline targeting.

Neuronavigation was performed with Brainsight (Rogue Research), co-registering participant MRIs with coil position. Average coil orientations are provided in Supplementary Table S1. iTBS was delivered using a MagStim D70 coil at 80% of resting motor threshold (RMT) or 52% maximum stimulator output, whichever was lower (Chung et al., 2018; Hoy et al., 2016). Due to hardware limitations of the TMS unit, 52% was the highest possible power to deliver a full iTBS sequence. Therefore, participants received the lesser of either 80% RMT or 52% machine output. Out of the 61 sessions, 34 of them had to use the 52% power threshold, which were evenly distributed across the three stimulation sites: L-FPN-B = 11 sessions, Vertex = 12 sessions, and D-FPN = 11 sessions. The average RMT was also consistent across the three sites: L-FPN-B = 66.26% (SD=8.1%), Vertex = 67.36% (SD=9.64%), and D-FPN = 68.05% (SD=9.51%), with a grand average of 67.21% (SD=9.05%).

### 2.7 Behavioral data processing and modeling

Trials with response times less than 0.2 seconds and over 1.5 seconds were excluded. For each participant, session, and task condition (e.g., Stroop High Conflict vs. Low Conflict), we first fit a drift diffusion model (DDM) with three free parameters (drift rate, threshold, non-decision time) using the fast-dm library (Voss & Voss, 2007). We selected fast-dm because it natively implemented the Kolmogorov-Smirnov (KS) optimization criterion. We used the Kolmogorov-Smirnov (ks) method with a precision set to 5.0, as this implementation is robust to fast-contaminant outliers and provides high-precision estimates of the drift rate with our trial counts (Lerche et al., 2017). In contrast to Bayesian sampling approaches, fast-dm identifies best-fitting parameter values through a multidimensional search using the Nelder-Mead simplex algorithm, choosing the parameter values that minimize the KS statistic. Consequently, this approach does not require a priori assumptions regarding candidate value distributions. Following guidelines for maximizing parameter stability (Voss et al., 2015), we chose a parsimonious model where inter-trial variabilities were set to zero (sv = 0, szr = 0, st0 = 0), differences in non-decision time were set to zero (d = 0), and the starting point was set to be unbiased (zr = 0.5). Crucially, the diffusion noise coefficient is set to 1 in fast-dm (Voss & Voss, 2007), which is mathematically necessary to ensure parameter identifiability (Nunez et al., 2025). To verify the stability of these estimates and ensure the model was properly constrained, we performed a parameter recovery procedure (Supplementary Fig. S2) and posterior predictive checks (Supplementary Fig. S1). Detailed Methodology and recovery results are provided in the Supplementary Materials under “Model Fit Validations and Parameter Recovery”.

**Fig. 3.**
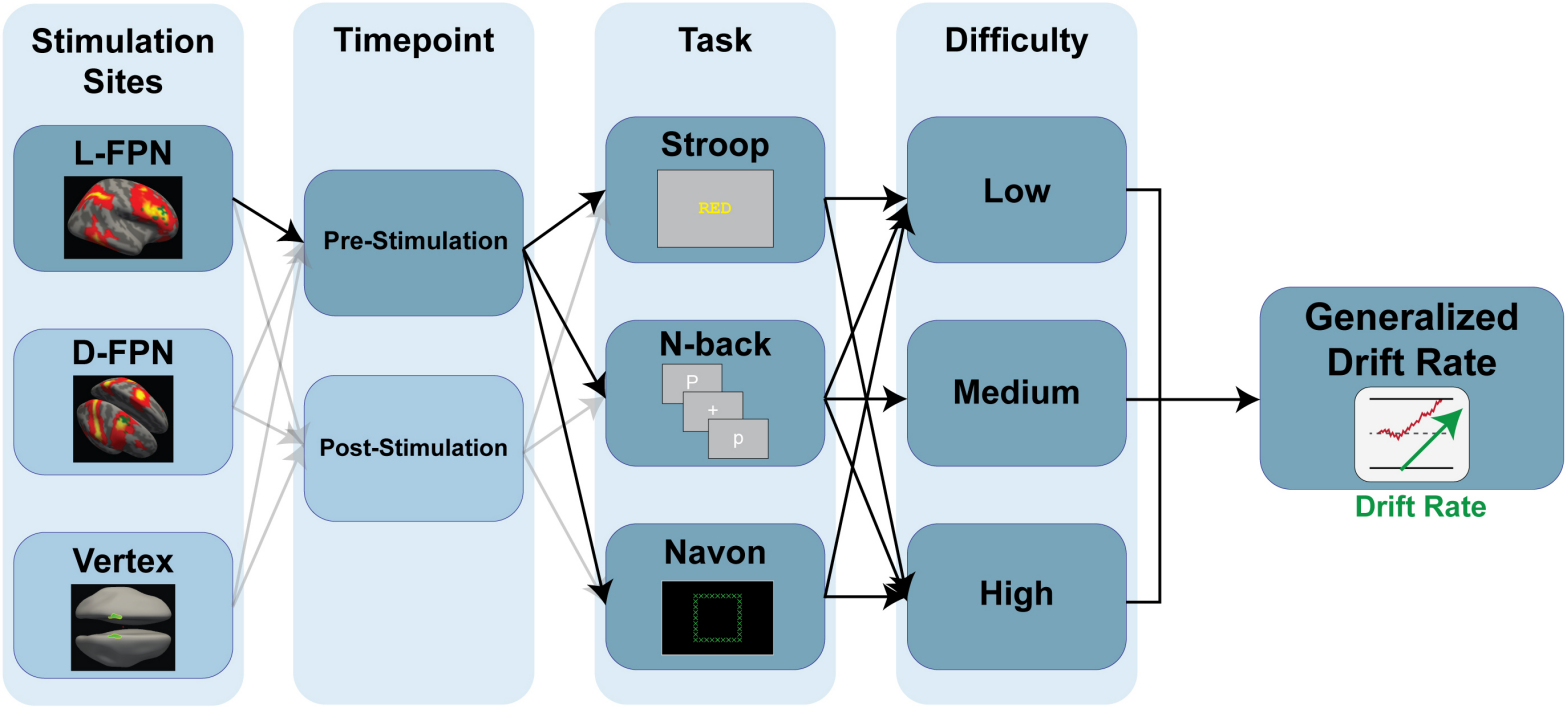
Modeling Pipeline for Generalized Drift Rate Extraction. Behavioral data were first partitioned by Stimulation Site and Timepoint. The highlighted path illustrates a single generalized drift rate calculation for a participant (e.g., L-FPN Stimulation Site at the pre-stimulation timepoint). Within this path, a drift diffusion model (DDM) was fit for every combination of task (Stroop, N-back, and Navon) and difficulty levels (Low, Medium, and High), yielding multiple drift rate estimates for a single path. These multiple drift rates were used to derive the Generalized Drift Rate in a single-factor confirmatory factor model. As indicated by the faded sections, this process was repeated across all combinations of the three stimulation sites and two timepoint, resulting in up to six distinct generalized drift rates per participant for the final mixed-effects analysis.

To derive a task-general measure of cognitive control performance, we estimated a single-factor confirmatory factor model across the individual drift rates from all three tasks and difficulty conditions, informed by the factor-analytic framework of Weigard et al. (2021). We favored this latent approach over traditional difference scores (e.g., high minus low conflict) to avoid the low reliability associated with subtraction metrics, detrimental to individual-difference analyses. We prioritized this method to ensure high sensitivity to individual differences in cognitive control capacity, acknowledging a potential tradeoff with specificity regarding conflict-level adjustments. Exploratory factor analysis with parallel analysis (Horn’s method, 1,000 iterations) indicated that although two components exceeded the empirical threshold of 1 (1.32 and 1.01 respectively), the second component was marginal (Fig. 6). The resulting latent factor, which we refer to as the generalized drift rate, was estimated separately for each participant for every combination of stimulation site and stimulation timepoint (pre- and post-stimulation). The model was estimated using robust maximum likelihood (MLR) with full information maximum likelihood (FIML) to account for missing data, as not all participants completed every session. Standardized factor loadings and fit indices were used to assess model adequacy. All factor analyses were performed in R (version 4.2.2) using the *psych* and *paran* packages for exploratory analysis and *lavaan* for confirmatory analysis. Following model estimation, we extracted these individual-level factors to serve as the primary outcome measure in our downstream linear mixed-effects models. The full DDM processing pipeline is shown in Figure 3.

### 2.8 Statistical analysis

As preliminary checks, standard paired t-tests were used to confirm the expected effects of task difficulty on raw performance metrics (response time, accuracy) and task-specific drift rates. To ensure that counterbalancing was effective and that no systematic baseline differences existed between stimulation sessions, we conducted one-way repeated-measures ANOVAs comparing the pre-stimulation drift rates across the three stimulation sites for each task.

We then employed a linear mixed-effects model to examine variables affecting the generalized drift rate. As measurements were obtained from multiple sessions for the same individual, these measurements are not independent and violate the assumptions of a standard linear regression (James et al., 2021). We, therefore, used a multilevel model with maximum-likelihood estimation to account for hierarchical structures within our data (Raudenbush & Bryk, 2002). Consequently, the t-values reported for the generalized drift rate outcomes represent the t-statistics from the fixed-effect estimates from these models, rather than separate t-tests.

The baseline model tested whether L-FPN/M-FPN anti-correlations predicted generalized drift rate. An intermediate model assessed the effects of TMS on generalized drift rate without considering network interactions. The main model then tested whether L-FPN/M-FPN anti-correlations predicted TMS-induced changes in the generalized drift rate and whether these effects interacted with the stimulation site (L-FPN-B, D-FPN, and Vertex).

We were not able to use the maximal model (Barr et al., 2013), as the random effects structure was too complex and did not converge. We were only able to add the random intercepts, as including additional terms prevented the model from converging: leading to the following random effects structure in our main model:

$$\begin{array}{l} v\;\sim \;L-FPN/M-FPN\;*\;Site*\;Stimulation\\ \;+Days+(1\;|\;Participant) \end{array}$$

where *v* represents the generalized drift rate, *L-FPN/M-FPN* represents the between-network connectivity of the L-FPN and M-FPN, *Site* represents the location of stimulation (L-FPN-B, D-FPN, or vertex), *Stimulation* is a binary variable indicating whether the trial was done before or after stimulation, and *Days* is an additional covariate indicating the number of days since the first stimulation session. *Participant* is a categorical variable indicating the participant ID.

All models used the bobyqa optimizer to ensure convergence. The random effects structure accounted for estimation bias due to unequal and small sample sizes. None of the final models reported convergence warnings. Additionally, to ensure that baseline head motion during the MRI scan did not confound our observed relationships, we re-ran these models including mean framewise displacement (FD) as a covariate. The results remained qualitatively unchanged (see Supplementary Materials for details).

Finally, to identify which specific pairs of stimulation sites drove observed rTMS effects, we conducted post-hoc interaction contrasts using the emmeans (version 1.7.2) package. Because standard contrast functions inconsistently marginalize continuous covariates in interactions, we first residualized the effect of ‘Days’ from the generalized drift rate. By performing our pairwise site comparisons on these residuals, we ensured that the contrasts accurately isolated the stimulation effects from any practice-related variance.

## 3 Results

### 3.1 Summary statistics

#### 3.1.1 Raw metrics (RT and accuracy)

In the Stroop task, mean accuracy was 97.9% (SD = 1.2%) with a mean response time (RT) of 713 ms (SD = 79 ms). Paired t-tests showed that RTs increased from the no-conflict condition (M = 693 ms, SD = 80 ms) to the high-conflict condition (M = 732 ms, SD = 84 ms; p = .001). Similarly, accuracy decreased from the no-conflict condition (M = 98.1%, SD = 1.1%) to the high-conflict condition (M = 97.6%, SD = 1.4%; p = .028).

In the Navon task, mean accuracy was 97.1% (SD = 1.5%) with a mean RT of 850 ms (SD = 69 ms). Paired t-tests showed that RTs slowed from the no-switch condition (M = 798 ms, SD = 76 ms) to the switch condition (M = 1006 ms, SD = 65 ms; p < .001). Accuracy also declined from the no-switch condition (M = 97.8%, SD = 1.4%) to the switch condition (M = 95.2%, SD = 2.3%; p < .001).

In the N-Back task, mean accuracy was 95.9% (SD = 2.8%) with a mean RT of 508 ms (SD = 93 ms). A repeated-measures ANOVA showed a significant main effect of working memory load on both RT (p < .001) and accuracy (p < .001). RTs increased from 0-back (M = 423 ms, SD = 77 ms) to 1-back (M = 501 ms, SD = 102 ms) to 2-back (M = 609 ms, SD = 145 ms), while accuracy decreased from 0-back (M = 97.8%, SD = 2.3%) to 1-back (M = 95.7%, SD = 2.3%) to 2-back (M = 94.0%, SD = 5.2%).

**Fig. 4.**
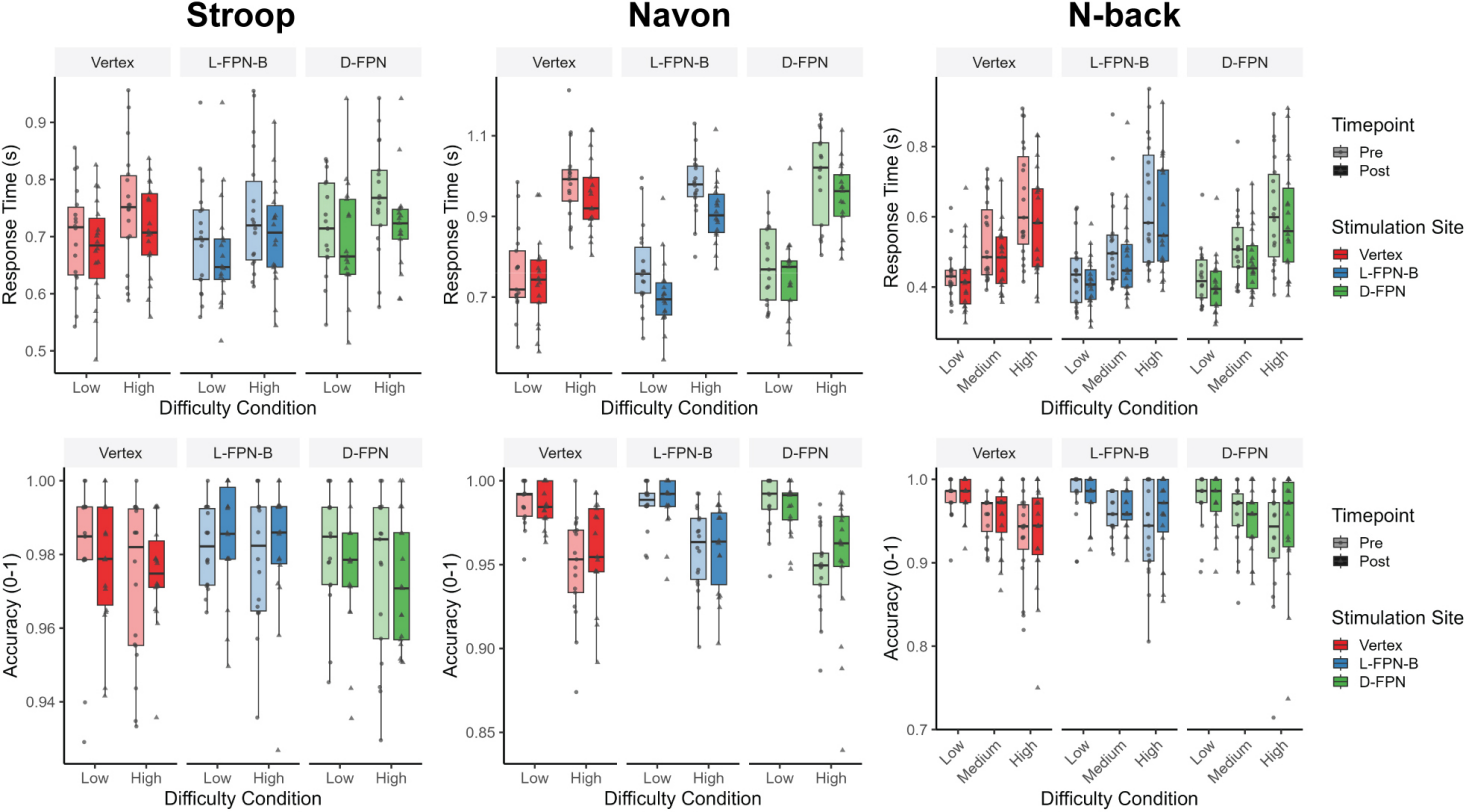
Raw Behavioral Performance Metrics Across the Stroop, Navon, and N-back tasks. The top row shows mean reaction times (ms), while the bottom row illustrates mean accuracy rates (0–1 scale). Each plot is separated by difficulty condition, stimulation site (Vertex, L-FPN-B, D-FPN), and timepoint (Pre vs. Post Stimulation). Boxplots represent the median and interquartile range for each condition, with individual participant data points overlaid. A consistent pattern is evident across all three tasks, where increasing difficulty resulted in significantly longer reaction times and lower accuracy.

Data plots with all raw mean performance metrics are displayed in Figure 4.

#### 3.1.2 Drift rate (task and condition specific)

For more reliable and interpretable results, we computed drift rates (v) across the tasks. Our drift rate results were qualitatively similar to those of the raw task metrics, showing that higher difficulty corresponded to lower drift rates.

In the Navon task, mean drift rate was 2.87 (SD = 1.39). Paired t-tests showed that participants had a significantly lower drift rate (p < .001) in the switch condition (M = 2.26, SD = 1.10) compared to the no-switch condition (M = 3.47, SD = 1.38).

In the N-Back task, mean drift rate was 3.64 (SD = 1.91). A repeated-measures ANOVA showed a significant main effect of working memory load on drift rate (p < .001). Drift rates decreased from 0-back (M = 5.09, SD = 1.95) to 1-back (M = 3.40, SD = 1.52) to the 2-back (M = 2.41, SD = 1.12).

In the Stroop task, mean drift rate was 2.77 (SD = 1.20). Paired t-tests showed that participants had a trending lower drift rate (p = .09) in the conflict condition (M = 2.63, SD = 1.18) compared to the no-conflict condition (M = 2.91, SD = 1.20).

**Fig. 5.**
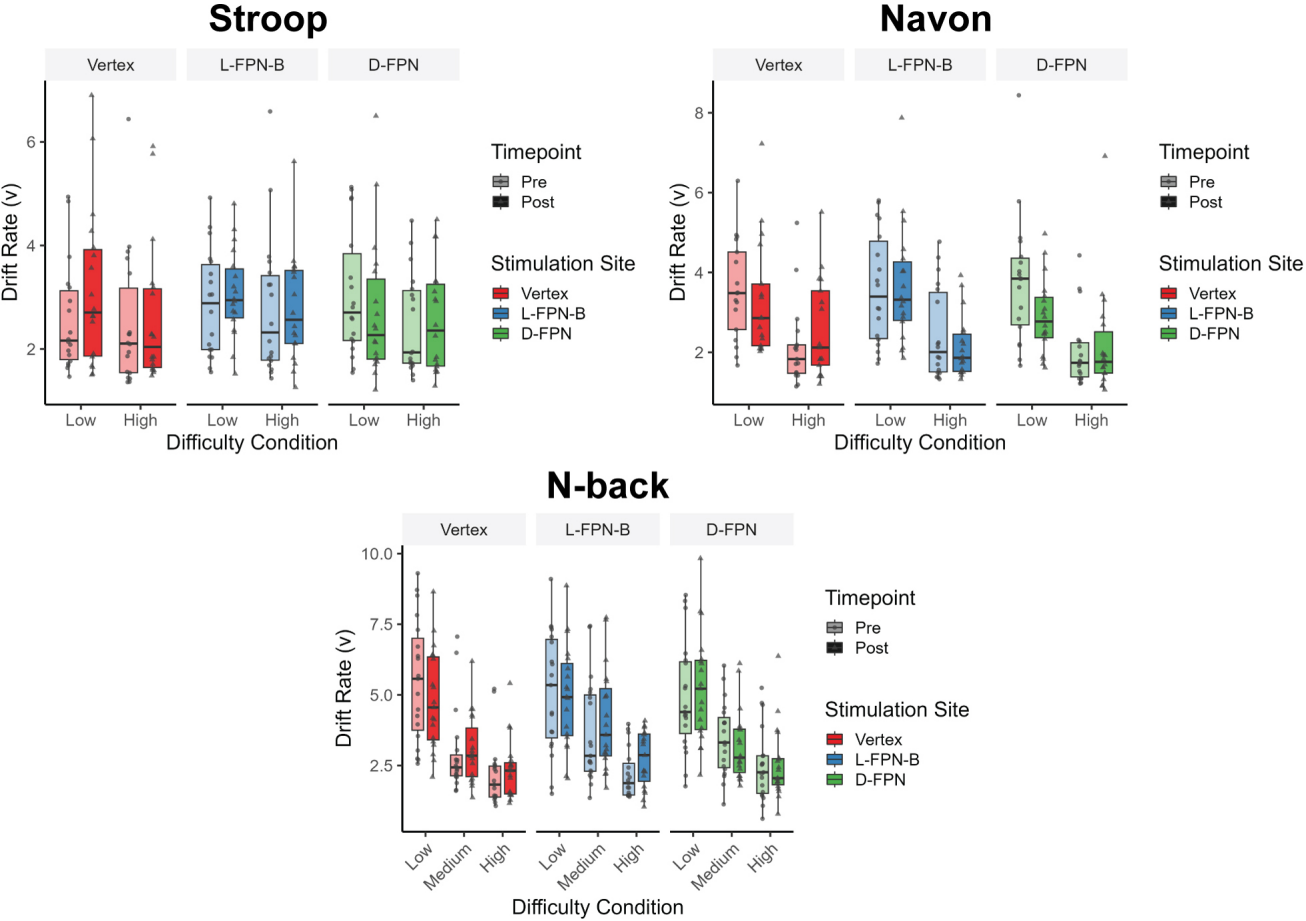
Drift rate (v) estimates across the Stroop, Navon, and N-back tasks. Each plot shows the distribution of drift rates separated by difficulty condition, stimulation site (Vertex, L-FPN-B, D-FPN), and timepoint (Pre vs. Post Stimulation). Boxplots represent the median and interquartile range, with individual participant data points overlaid. Consistent with the raw behavioral metrics, there is a significant decrease in drift rates as task demands increased across all three paradigms.

Data plots with all mean drift rate performance metrics are displayed in Figure 5.

### 3.2 Validation of factor structure

**Fig. 6.**
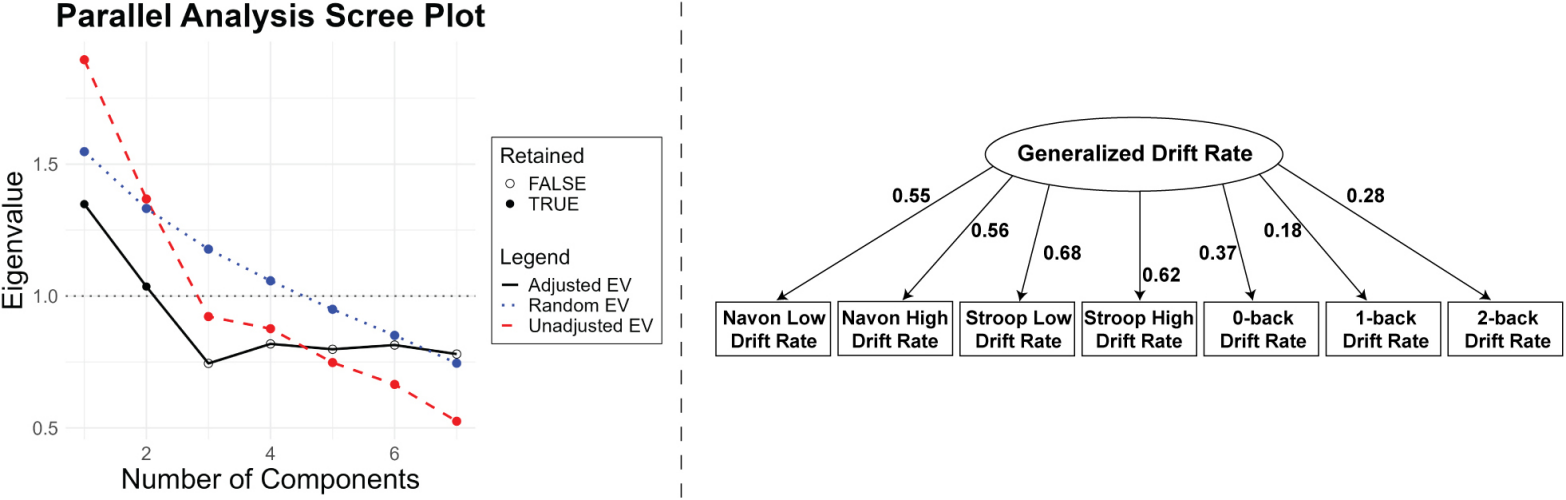
Factor Analysis on Drift Rate. Left: Scree plot from parallel analysis. Components to be retained are based on unadjusted eigenvalues from observed data (red line) exceeding those from random data (blue line). Adjusted eigenvalues (black line) confirm this, with two components above 1 (1.35 and 1.04 respectively). Despite the marginal emergence of a second factor, a one-factor solution was retained to maintain a parsimonious, task-general index of cognitive control. Right: Standardized factor loadings from a one-factor CFA model. Loadings vary across tasks, with Navon and Stroop showing the strongest loadings.

To integrate these task-specific metrics into a unified measure of cognitive control performance, we utilized factor analysis to extract a latent generalized drift rate from the individual drift rate parameters computed across the three tasks. Exploratory factor analysis indicated that although two components exceeded the empirical threshold of 1 (1.35 and 1.04 respectively), the second component was marginal (Fig. 6). To prioritize a task-general index of cognitive control, we retained a one-factor solution representing the generalized drift rate, aligning with previous factor analyses that show the dominance of this first factor (Weigard et al., 2021). Accordingly, we conducted a one-factor confirmatory factor analysis (CFA) representing the generalized drift rate. This initial model showed poor fit: the scaled chi-square test was significant, χ²(14) = 27.53, p = .016, Comparative Fit Index (CFI) = 0.747, Tucker-Lewis Index (TLI) = 0.620, Root Mean Square Error of Approximation (RMSEA) = 0.086, Standardized Root Mean Square Residual (SRMR) = 0.077. Loadings were generally high, except for the n-back conditions. Removing the 1-back, which had the worst fit (p = .51), improved fit substantially, χ²(9) = 9.96, p = .354, along with CFI = 0.984, TLI = 0.973, RMSEA = 0.024, SRMR = 0.049. Despite the improved fit, the factor loadings and results were consistent across models, leading us to retain the full model for completeness.

### 3.3 Baseline results

Baseline L-FPN/M-FPN connectivity did not significantly predict pre-stimulation generalized drift rate (t_19.00_ = -1.145; p = .27), although the trend suggested that greater integration related to poorer performance. Furthermore, a series of one-way repeated-measures ANOVAs confirmed there were no significant differences in pre-stimulation drift rates across the three sessions for all tasks (F(2, 124) = 0.456, p = .635), or for the individual Stroop (p = .16), N-back (p = .58), or Navon (p = .84) tasks. These findings indicate that the variations in baseline performance likely reflect sampling noise rather than systematic session differences. Days since the first session did not significantly predict baseline drift rates (t_44.09_ = 1.43; p = .16), although the trend was consistent with practice effects. Consequently, these results indicate that baseline connectivity did not significantly influence drift rate.

### 3.4 Raw stimulation results

**Fig. 7.**
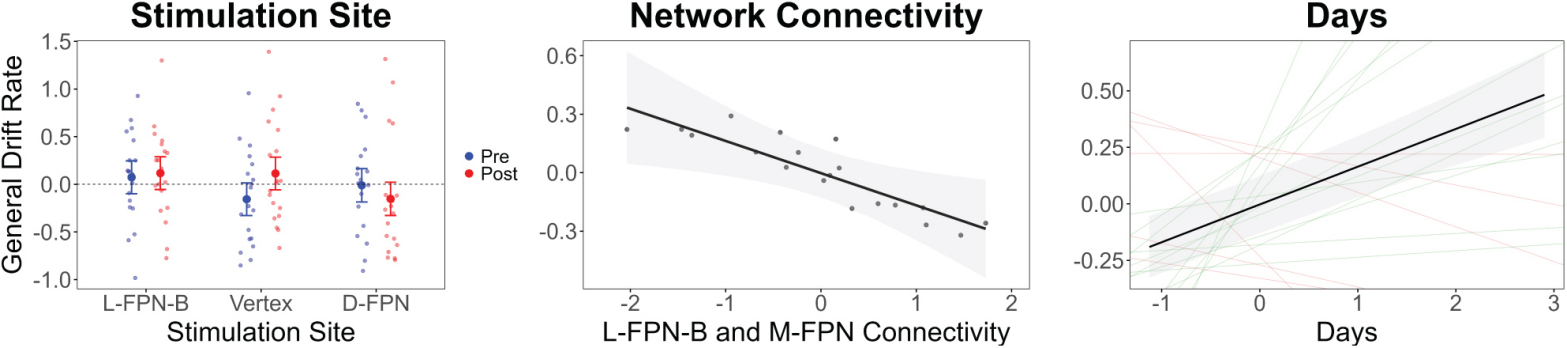
Effects of Various Predictors on Generalized Drift Rate. The figure shows the estimated general drift rate as a function of (Left) Stimulation Site and Timepoint, (Middle) L-FPN-B and M-FPN Connectivity, and (Right) Days since first stimulation session. *Left Panel*: Large points show estimated marginal means for each condition, calculated by holding L-FPN-B/M-FPN connectivity and the “Days” covariate at their sample means. Bars represent standard errors, and the small points show individual participant averages, centered by removing individual-specific random intercepts to account for the mixed effects model output. *Middle Panel*: The solid line represents the estimated marginal main effect of L-FPN-B/DMN connectivity, collapsed across all stimulation sites and timepoints, while holding “Days” at its sample mean, with a one standard error gray ribbon. Points represent individual subject averages, also centered by removing random intercepts. *Right Panel*: The black line shows population-level estimated trends, averaged across all sites and timepoints, while holding connectivity at its sample mean, with a one standard error gray ribbon. Thin lines represent individual participant trends colored based on whether they were rising (green) or falling (red).

Without considering network moderators (Fig. 7), TMS did not significantly alter drift rate (t_94.99_ = 1.51; p = .13), nor did stimulation effects differ between the L-FPN-B and vertex sites (L-FPN-B-Site:Stimulation; t_94.99_ = -0.90; p = .37). However, the days covariate was significant (t_101.71_ = 2.73; p = .007) reflecting practice effects. Post-hoc contrasts also indicated no differences in stimulation effects between L-FPN-B and D-FPN sites (t_105_ = 1.44; p = .32). These null effects of stimulation may mask individual variability in network connectivity, supporting the necessity of the following moderation analysis.

### 3.5 Network moderators of stimulation

**Fig. 8.**
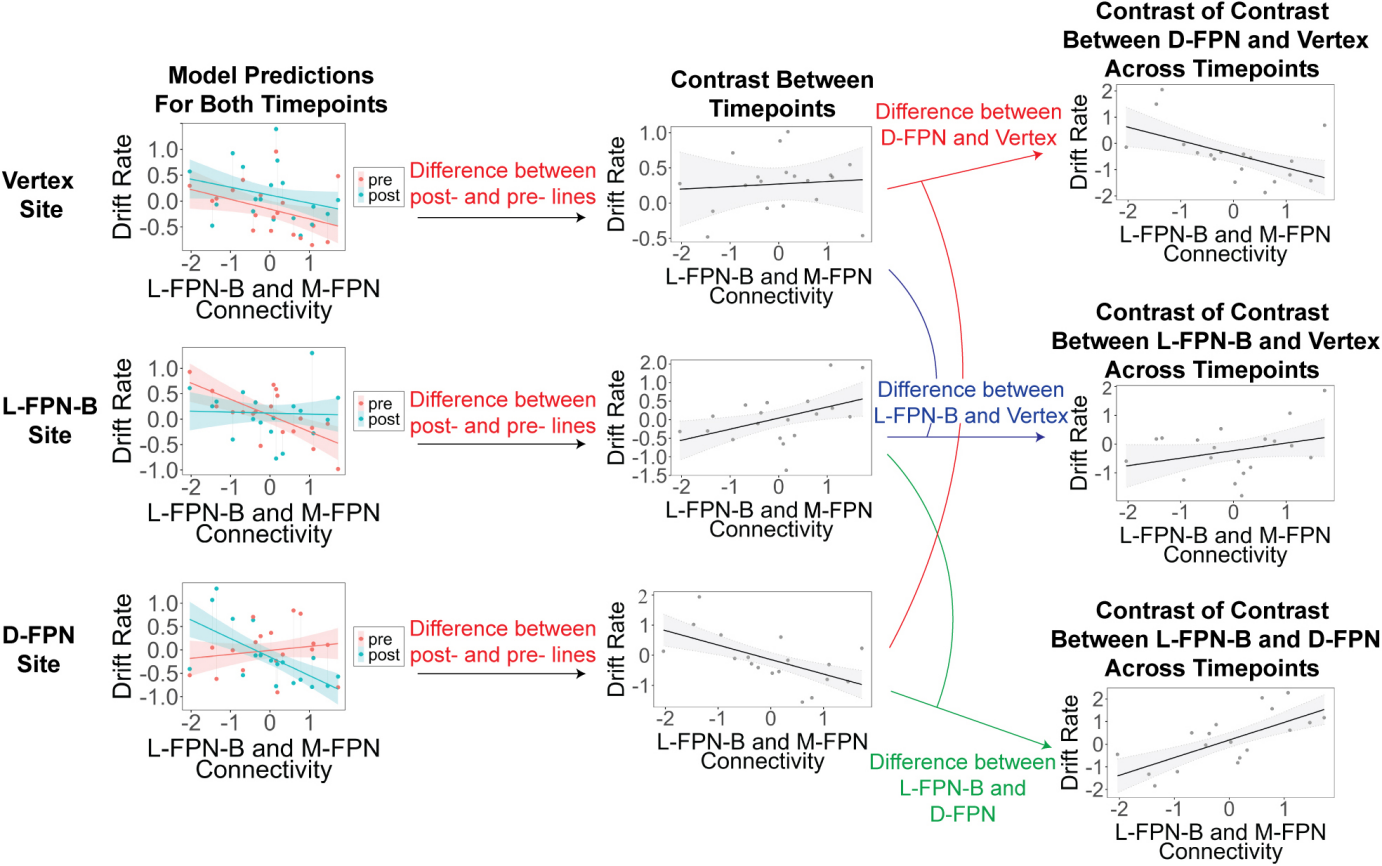
Network Interactions on TMS Effects for General Drift Rate. The first column shows the relationship between L-FPN-B:M-FPN Connectivity and General Drift Rate, split by stimulation site (L-FPN-B, Vertex, and D-FPN) and timepoint (pre- and post- stimulation). Individual points represent participant values centered by removing random intercepts to account for the mixed-effects model output, with gray lines connecting Pre and Post observations for each participant. Across conditions, greater integration between the L-FPN-B and M-FPN is associated with lower baseline drift rates. The second column shows the TMS-induced changes (post- minus pre-) as a function of connectivity. Solid points represent within-participant difference scores. While the vertex trend is flat, L-FPN-B stimulation shows a positive slope, indicating that individuals with more integrated L-FPN-B and M-FPN networks experience larger performance improvements (higher drift rates) after stimulation. The third column contrasts the stimulation sites directly. The bottom-right panel shows that for individuals with high L-FPN-B and M-FPN network integration, L-FPN-B stimulation results in significantly higher drift rates compared to D-FPN stimulation. Across all panels, solid lines represent model estimates and shaded regions indicate standard errors of the mean.

We next tested whether L-FPN/M-FPN connectivity moderated stimulation effects (Fig. 8). The main model showed no overall TMS effect on drift rate (t_94.98_ = 1.60; p = .11), no interactions of stimulation with site (L-FPNB-Site:Stimulation; t_94.98_ = -0.957; p = .34; D-FPN-Site:Stimulation; t_94.98_ = -1.74; p = .65), or interactions of stimulation presence with baseline connectivity (t_94.97_ = -.16; p = .09). However, connectivity showed a significant interaction with stimulation at the D-FPN site compared to the vertex site (t_94.98_ = -2.23; p = .028), with greater network segregation predicting higher drift rates after D-FPN stimulation. Post-hoc contrasts confirmed that connectivity had a significant interaction for L-FPN-B vs. D-FPN stimulation (t_106_ = 3.19; p = .0019). Individuals with more integrated networks showed higher drift rates after L-FPN-B compared to after D-FPN stimulation. This effect reflects opposite moderation effects relative to the vertex site: the connectivity interaction with L-FPN-B stimulation was positive relative to vertex (t_106_ = 1.08; p = .28), whereas the interaction for D-FPN stimulation relative to vertex was significantly negative (t_106_ = -2.11; p = .038). Together, these results indicate that higher L-FPN/M-FPN integration predicted improved drift rates after L-FPN-B stimulation, while higher L-FPN/M-FPN segregation predicted improved drift rates after D-FPN stimulation relative to the vertex.

## 4 Discussion

This study examined whether baseline L-FPN/M-FPN connectivity moderated TMS effects on cognitive control. Using generalized drift rate as a latent behavioral measure of task-general cognitive control, we found that baseline connectivity did not directly predict performance, and that stimulation of a single site did not enhance performance. However, baseline connectivity moderated stimulation outcomes: individuals with more integrated L-FPN/M-FPN networks benefited from L-FPN-B stimulation, whereas those with more segregated networks benefit from D-FPN stimulation. These findings highlight network dynamics as a basis for individualized TMS interventions and supported generalized drift rate as a sensitive behavioral index of cognitive control beyond traditional measures like response-time and accuracy.

Our results show that L-FPN/M-FPN interactions shape whether TMS enhances or disrupts cognitive control. Mind wandering suppression, as supported by L-FPN/M-FPN anticorrelations, is viewed as important for cognitive control to maintain optimal task focus (Godwin et al., 2017; van Son et al., 2019). The L-FPN is thought to regulate M-FPN suppression by coordinating shifts between the internally oriented M-FPN and the externally focused D-FPN (Cole et al., 2013; Dixon et al., 2017). In particular, certain nodes of the L-FPN function as flexible hubs that coordinate these network shifts by flexibly decoupling from the M-FPN to couple with the D-FPN during externally-oriented tasks (Dixon et al., 2018). We view the baseline connectivity between the L-FPN and M-FPN as a trait-like indicator of an individual’s readiness associated with this reconfiguration. In this framework, individuals with more integrated baseline L-FPN/M-FPN connectivity gained the most from L-FPN stimulation, suggesting that TMS promoted the necessary decoupling of the L-FPN from the M-FPN to promote external focus. By contrast, D-FPN stimulation benefited individuals with already well-segregated L-FPN and M-FPN networks. For these individuals, additional decoupling is likely not needed and performance gains came from directly stimulating external attention systems.

Interestingly, baseline L-FPN/M-FPN coupling did not predict performance in the absence of stimulation. This null finding may reflect limited statistical power or noise in task engagement, but it also suggests certain connectivity effects emerge most clearly when the system is perturbed (Douw et al., 2020; Godwin et al., 2017). Baseline connectivity may also support control through different task-specific pathways that offset each other at the group level or reflect individual differences in cognitive strategy (Braver, 2012; Purg Suljič et al., 2024). Together, these results show that stimulation effects depend on both stimulation site and baseline connectivity, and that null effects in prior TMS studies may reflect averaging across individuals with opposing network configurations (Dengler et al., 2024; C. J. Lowe et al., 2018). It is important to note that the individual differences observed in baseline L-FPN/M-FPN integration could be fundamentally driven by both genetic and developmental factors. Twin studies have demonstrated that both static and dynamics network properties are heritable (Barber et al., 2021; Xu et al., 2017). Furthermore, this network architecture changes across the lifespan, with the L-FPN/M-FPN becoming more segregated during development (Chen et al., 2023) and then toward increased integration during healthy aging (Geerligs et al., 2015). These biologically rooted differences suggest that baseline integration is a robust, trait-like biomarker for TMS effectiveness in cognitive control, offering a stable target for personalized interventions.

An important strength of the current study is the use of latent variable modeling with computational model parameters to characterize cognitive control, offering a reliable alternative to traditional reaction-time and accuracy based metrics (Friedman & Miyake, 2017; Von Bastian et al., 2020). Our latent factor approach modeled performance across demand levels, capturing common variance while reducing task impurities and measurement error (Eisenberg et al., 2019; Friedman & Miyake, 2017). The generalized drift rate further removes non-decision processes, such as sensorimotor processes, and quantifies the efficiency of evidence accumulation (Forstmann et al., 2016). Importantly, higher drift rates can arise from multiple cognitive control domains, such as stronger inhibition, working memory, or flexibility, all of which enhance decision-relevant signal-to-noise. Thus, while the generalized drift rate removes task-specific variance, it can still meaningfully reflect core cognitive control mechanisms. Nonetheless, this approach may obscure load-dependent differences in control engagement that are often measured with difference scores. Although difference scores are less reliable, comparing drift rates between low- and high-demand conditions can provide valuable information about how individuals scale cognitive control with increasing demands. Future work should, therefore, consider both aggregated and load-dependent approaches, as each may highlight different aspects of cognitive control.

Our factor analysis results contribute to the broader debates over whether cognitive control is unitary, multidimensional, or hierarchical (Von Bastian et al., 2020). Our exploratory factor analysis (EFA) identified a secondary component, supporting the view that cognitive control may be organized hierarchically, with a general component and task-specific elements similar to proposed structures of intelligence (Schneider & McGrew, 2018). However, we favored a one-factor solution in our analysis to isolate the common variance associated with cognitive control across tasks. While larger studies use bi-factor models to separate the general and task-specific components (Friedman & Miyake, 2017), the general factor usually dominates. For example, although Weigard et al. (2021) found a bifactor structure for drift rates, the general factor explained 82% of the total variance. Given our sample size, focusing on the single, robust common factor provided a more stable basis for our conclusions (Kyriazos, 2018; Lorenzo-Seva & Ferrando, 2024), whereas the secondary component likely reflects narrower task-specific performance nuances that fall outside the current study’s scope. The generalized drift rate component identified here aligns with prevailing interpretations of a common cognitive control factor, reflecting goal maintenance and distractor suppression (Friedman & Miyake, 2017; Von Bastian et al., 2020). Our results support this view by showing that changes to this common factor through TMS depend on baseline L-FPN/M-FPN connectivity, a network interaction thought to support internal distraction suppression and goal-directed cognition (Douw et al., 2016; Maillet et al., 2019). Importantly, the ability to predict and influence this general drift rate factor highlights its promise as a psychometrically robust target for neuromodulation.

Several limitations and future directions warrant consideration. First, the absence of task-based fMRI limits our ability to determine whether the resting-state connectivity findings extend to task contexts. Block-level analyses following stimulation could directly link connectivity changes to DDM parameters, clarifying whether behavioral improvements reflect shifts in control network dynamics. Second, larger samples with denser within-subject designs are needed to improve statistical power, identify latent factor structures, and test the stability of baseline drift rates and stimulation effects over time. Another common challenge in smaller samples is variability in baseline performance, evident in our study. Despite counterbalancing, pre-stimulation D-FPN sessions showed lower performance, and the association between performance and L-FPN/M-FPN connectivity was slightly positive, unlike the negative trends in the other pre-stimulation sessions, likely reflecting sampling noise that larger datasets could mitigate. Crucially, the impact of DAN stimulation was robust enough to overcome this initial positive baseline, driving a steep negative relationship post-stimulation that exceeded the magnitude of the vertex control. This reversal suggests that the site-specific stimulation effect was powerful enough to overcome baseline sampling noise and produce a robust relationship.

Beyond baseline variability, repeated measures of cognitive control are inherently susceptible to practice effects. Performance improvements over time are a potential confound because participants completed the same tasks across multiple visits and within the same session. While we mitigated these effects experimentally through the counterbalancing of stimulation sites and analytically by including the time between sessions as a covariate, practice-related noise remains a challenge in multi-session designs. For instance, the pre-to-post performance increase observed in our vertex condition suggests that a within-session practice effect may be present. Future research could minimize these confounds by training participants to perform near their ceiling before the first stimulation session, or by employing alternate psychometrically matched tasks.

In this study, we used generalized drift rate as a robust latent behavioral measure of cognitive control, but it collapses multiple processes into a single parameter. Although this improves reliability over difference score metrics, it remains a relatively coarse measure, incorporating processes such as attentional allocation, threshold adjustments, and dynamic evidence accumulation. More advanced models that separate these processes (Krajbich et al., 2010; Weichart et al., 2020; White et al., 2011), combined with neuroimaging approaches such as fMRI or EEG, could yield a more precise account of cognitive control and improve the interpretability of model-based inferences.

## 5 Conclusion

Overall, our study suggests that baseline differences in resting-state functional connectivity shape how TMS influences cognitive control. Our findings suggest that participants with more integrated L-FPN/M-FPN networks benefit the most from stimulation to the L-FPN. In contrast, participants with more segregated L-FPN/M-FPN networks benefit the most from stimulation to the D-FPN. This study contributes to the growing literature revealing that stimulation outcomes are not uniform across individuals, and that personalized approaches to neuromodulation should consider baseline network configurations. Moreover, we confirm the utility of the generalized drift rate as a viable task-general, latent measure of cognitive control, aligning with theoretical accounts of a common control construct supported by dynamic interactions between the L-FPN and the M-FPN. Future work incorporating task-based neuroimaging, larger and more diverse samples, and more sophisticated neurocognitive models will be essential to develop individualized interventions for cognitive control.

## Supplementary Material

Supplementary Material

## Data Availability

The R Code and raw data files required to reproduce the analyses reported are available at: https://github.com/CogNeW/project_L-FPN_M-FPN_cc_stim
