# Supplementary material for: Frontoparietal control-default mode connectivity predicts TMS effects on cognitive control
Source: Imaging Neurosci (Camb). 2026 Jul 14;4:IMAG.a.1298. doi: 10.1162/IMAG.a.1298 (PMC13370750; doi:10.1162/IMAG.a.1298)
Supplement: Supplementary Material [file IMAG.a.1298_supp.pdf]

Supplemental Information

Methods

Task Specification

During each session, participants performed three computerized cognitive control tasks: the Navon, Stroop, and *n*-back. The Navon and Stroop tasks were administered on MATLAB R2021a through the Psychtoolbox Version 3.0.17, while the *n*-back was administered using Psychopy v2021.1.4.

Navon Task

The Navon task requires the participant to switch between local and global processing of visual stimuli (Navon, 1977). In each trial a larger global shape is shown that is composed of smaller local shapes. The local and global shapes were one of the four – a circle, X, triangle, or square. We asked participants to identify either the global shape when the stimulus was green, and the local shape when the stimulus was white. A fixation cross was displayed at the beginning of each trial for 0.1 seconds, while the stimulus appeared on the screen for 1.9 seconds. Every block consisted of 144 trials. Participants responded using the following right-handed keypresses: V for circle, B for X, N for triangle, and M for square.

There were two experimental conditions, a low demand (non-switching) condition and high demand (switching) condition. In the high condition, the stimulus color changed on half of the trials, forcing the participant to switch between local and global processing. On the low condition there was only one color switch, which occurred halfway through the block. Participants saw a brief instruction slide indicating that a color switch would occur on the next trial for the low demand condition. For the low demand condition, the starting color was counterbalanced across all participants. For each condition, we generated six versions of the trial order which were counterbalanced across participants.

Participants completed a 24-trial low and high demand practice block before starting the experimental blocks. Participants proceeded from the practice block if they achieved 50% accuracy. Participants unable to reach this accuracy threshold within three attempts of the practice blocks were excluded from analysis.

Stroop Task

The Stroop Task engages inhibitory processes by forcing the participant to suppress the automatic response of reading (MacLeod, 2005). Color words are printed with a matching or mismatching text color, and participants were asked to identify the text color. There were two experimental conditions, a low demand (no response conflict) and a high demand condition (response conflict). The low-demand condition consisted of non-color words that were matched to the color words for length (“far”, “deal”, “horse”, and “plenty”). The high-demand condition consisted of the colors words “red”, “blue”,

“green”, and “yellow” printed on a mismatching background text color. For each condition, we generated six versions of the trial order which were counterbalanced across participants.

A fixation cross was displayed at the beginning of each trial for 0.1 seconds, while the stimulus appeared on the screen for 1.9 seconds. Every block consisted of 144 trials. Participants responded using the following right-handed keypresses: V for red, B for green, N for yellow, and M for blue. Participants underwent the same practice block structure as described in the Navon section.

N-back

The *n*-back task is a standard executive working-memory task where participants decide whether a stimulus in a sequence matches the one that appeared *n* items ago. For this task, we tested three different values of *n*: 0, 1, and 2. For the 0-back condition, participants determined whether the current stimulus was an X.

Each condition consisted of 4 blocks with 17 trials per block. These four blocks were consistent across participants, but their order was counterbalanced across participants. Each stimulus appeared on the screen for two seconds or until the participant gave a response. Between each stimulus, a blank gray screen was presented for one second. The participant was to respond with the following right-handed keypresses: the right arrow key on a match, and the left arrow key on a non-match. Participants were informed that uppercase and lowercase should be treated equally. Participants underwent the same practice block structure as described in the Navon section.

Model Fit Validations and Parameter Recovery

Posterior Predictive Checks

To evaluate the fit of the drift diffusion model, we performed Posterior Predictive Checks (PPCs) for each of the three tasks. We generated synthetic datasets by simulating 100 trials for every set of DDM parameter estimates that were generated. We matched the simulated trial count to our actual trial counts to account for a similar level of sampling noise. The simulated trials were then aggregated across the entire sample to create a group-level posterior predictive distribution. These synthetic distributions separated by correct and error responses were then plotted against the observed data (Supplementary Figure 1).

The PPCs demonstrated that while the DDM provided an excellent fit for the N-back, discrepancies particularly for the error response time (RT) distributions were seen for the Stroop and Navon task. These tasks exhibited a flatter, bimodal error structure likely driven by automatic processes, such as the reading reflex in the Stroop or global shape precedence in the Navon. These automatic processes likely trigger faster errors not fully captured by constant drift rate models (Ulrich et al., 2015). This task-specific modeling artifact further supports our rationale for a latent factor approach. By extracting a generalized drift rate across three different tasks, we are isolating their shared variance to serve as a robust proxy for the common cognitive mechanisms that these tasks

demand, which include cognitive control functions such as goal maintenance and distractor suppression.

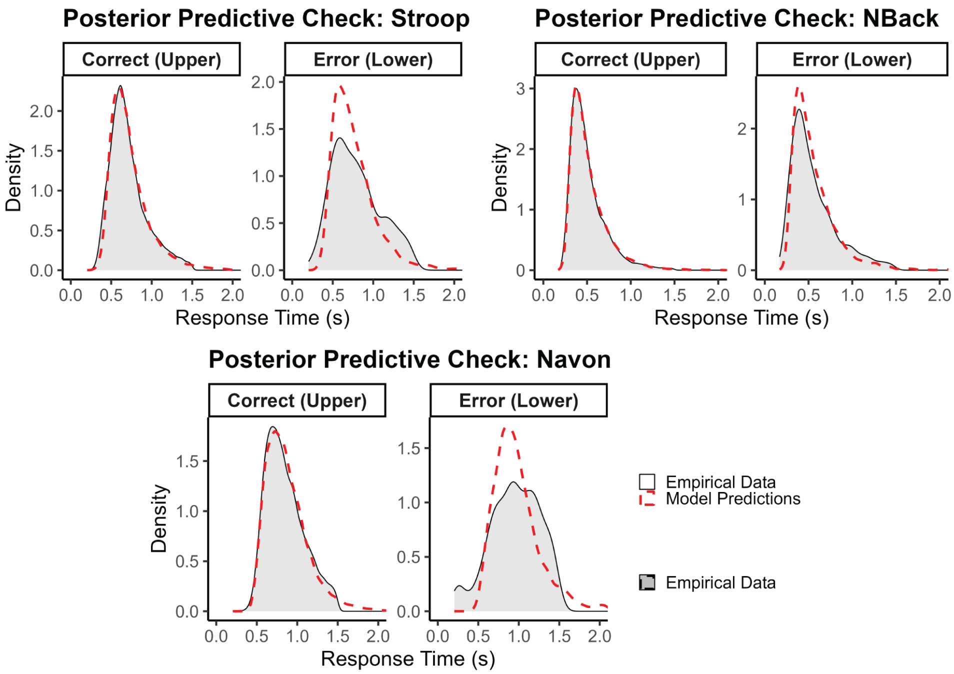

Supplementary Figure 1. Posterior predictive checks. Posterior predictive distributions (red dashed lines) are plotted against the empirical data (solid black lines) for the Stroop (left), N-back (center), and Navon (right) tasks. For each task, the distributions of Response Times are shown separately for Correct and Error responses.

Parameter Recovery

To verify the stability of our parameter estimates and ensure the drift diffusion model was properly constrained, we conducted a comprehensive parameter recovery analysis.

For each dataset, previously estimated parameters (drift rate, threshold, non-decision time) were used to generated synthetic dataset (n = 100 trials per condition) of response times and choices from a Weiner diffusion process. These simulated datasets were exported in fast-dm format and subsequently refit using the identical fast-dm estimation pipeline that was applied to the empirical data.

Parameter recovery was successful, showing strong Pearson correlations between the empirical and recovered estimates across all three tasks. Drift rate showed good recovery (r_stroop = .85; r_navon = .85; r_nback = .89). Non-decision time showed good recovery (r_stroop = .67; r_navon = .85; r_nback = .88), and threshold demonstrated good recovery (r_stroop = .62; r_navon = .75; r_nback = .73). These results are supported by scatterplots (Supplementary Figure 2) indicating strong linear fits and clustering along the diagonal for all parameters.

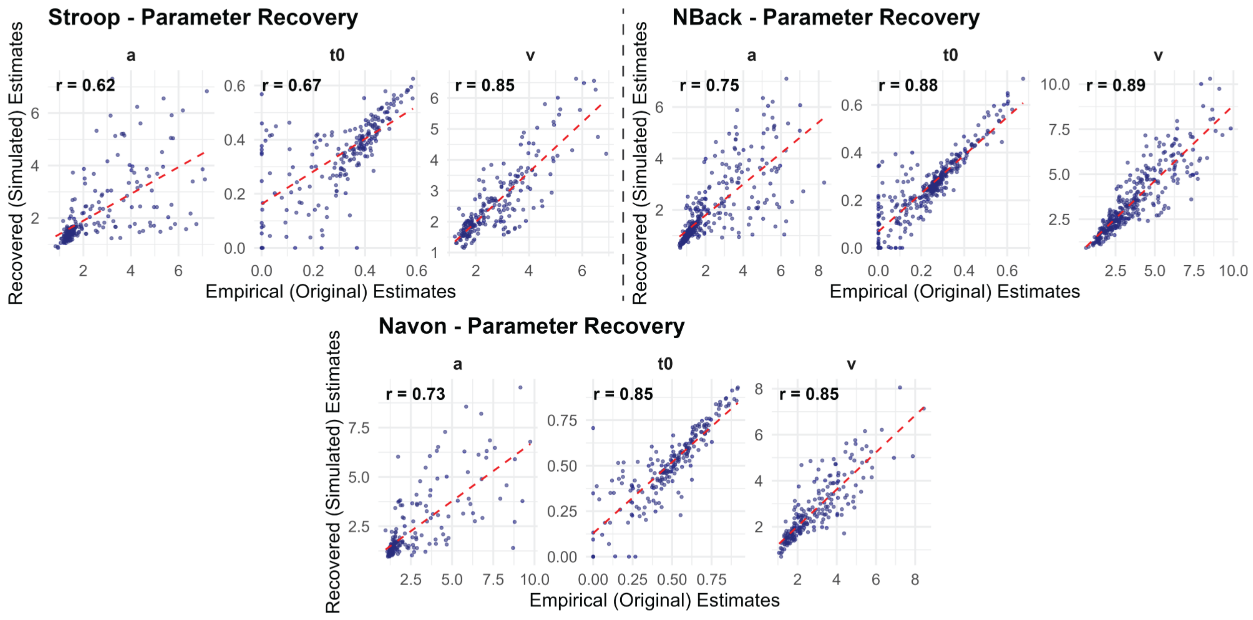

Supplementary Figure 2. Parameter recovery for the Stroop, N-back, and Navon tasks. Each panel displays the relationship between the original empirical parameter estimates (x-axis) and the recovered parameter estimates from the simulated data (y-axis) for boundary separation (a), non-decision time (t0), and drift rate (v). The dashed red line represents the line of best fit, demonstrating stable parameter recovery and appropriately constrained model fits across all three cognitive control tasks.

Brain Imaging Acquisition and Preprocessing

Acquisition

We acquired anatomical images and functional magnetic resonance images (fMRI) from a 3.0 Tesla Siemens Tim Trio whole-body scanner, using a whole-head elliptical coil and a single-shot gradient-echo T2* (TR=1,500 ms; TE=30 ms; flip angle = 60°; FOV = 19.2 cm; resolution 3mm x 3mm x 3mm). To ensure reliable within-participant estimation of functional networks, the participants completed a 30-minute resting-state scan.

Anatomical data preprocessing

We performed anatomical preprocessing with fMRIPrep 21.0.0 (Esteban et al., 2019). We corrected T1-weighted (T1w) images for intensity non-uniformity (INU), applied skull-stripping, and then performed tissue segmentation into cerebrospinal fluid (CSF), white matter (WM), and gray matter (GM). We created robust reference images from three separate T1w images (Reuter et al., 2010), which was used for brain surface reconstruction (Dale et al., 1999), reconciliation of multiple segmentation outputs (Klein et al., 2017), and volume-based spatial normalization (Fonov et al., 2011).

Functional Data Preprocessing

For each BOLD run per participant, we generated a reference volume and its skull-stripped version using fMRIPrep. We estimated head-motion parameters with FSL's mcflirt before applying any filtering and corrected slice timing with AFNI. We calculated a B0-nonuniformity map (fieldmap) from dual-echo GRE images to address

susceptibility distortions and co-registered the fieldmap to the BOLD reference. Using boundary-based registration, we aligned BOLD data to T1w images and resampled them onto both FreeSurfer surfaces and the native space.

We then computed confound time-series, including framewise displacement (FD) and DVARS, and extracted physiological noise components (CompCor) for noise correction. We flagged motion outliers and applied all transformations for head-motion correction, susceptibility distortion, and anatomical alignment in a single step. We used Lanczos interpolation to minimize smoothing during volumetric resampling and FreeSurfer tools for surface resampling. Finally, we used XCP engine to perform confound regression, remove physiological artifacts, and regress out task-based noise with FSL feat. Full pre-processing details can be found in Dengler et al. (2024).

Individualized Functional Network Mapping

We extracted functional networks using individualized parcellations, an approach that provides more precision and accuracy than group-average parcellations (Gordon et al., 2017; Lynch et al., 2020). We performed the iterative approach described and provided by Li et al. (2019), based on a prior approach by D. Wang et al. (2015). The parcellation returned a pairwise ROI-by-ROI matrix per individual containing Fisher z-transformed correlation coefficients, indicating the functional connectivity between each region pair (Biswal et al., 1995; Li et al., 2019; Lowe et al., 2000). We defined the between-network connectivity value as the average functional connectivity strength between each pair of regions between the FPCN and DMN. The within-network connectivity for each network was defined as the average functional connectivity strength between each pair of regions within that network and was defined separately for the FPCN and DMN.

Recent studies have indicated that the FPCN is heterogeneous and can be broken into two distinct subsystems with different roles, the FPCN-A and the FPCN-B (Dixon et al., 2018). The FPCN-A tends to couple with the DMN and is involved in the regulation of introspective processes, while the FPCN-B tends to couple with the dorsal attention network (DAN) and is involved in the regulation of perceptual attention. We chose to look solely at the FPCN-B for subsequent analyses, as the current project focused on FPCN and DMN interactions and their effects on TMS.

Likewise, the DMN consists of multiple interacting subsystems: a dorsal medial subsystem, a medial temporal subsystem, and a core hub region (Andrews-Hanna et al., 2014). The dorsal medial subsystem activates when mentalizing, inferring the mental states of others and oneself. The medial temporal system activates when constructing mental simulation and is crucial for episodic and spatial memory. The core hub system activates when information is thought to be personally significant and may update personal views to guide thoughts and behaviors. Out of the three subsystems, the core hub system has the most consistent and strong anticorrelations with task-positive networks (Dixon et al., 2017). Therefore, we focused on the DMN-core hub subsystem for the subsequent analyses.

Stimulation Site	Average Coil Orientation (m0, m1, m2)	Standard Deviation of Coil Orientation (m0, m1, m2)
**FPCNB**	m0: [0.3918, 0.1181, -0.8765]<br>m1: [-0.3917, 0.8898, -0.0568]<br>m2: [0.8197, 0.3598, 0.4055]	m0: [0.079961, 0.233572, 0.057734]<br>m1: [0.091342, 0.054059, 0.200964]<br>m2: [0.079989, 0.086010, 0.142599]
**DAN**	m0: [0.6617, 0.4495, -0.2884]<br>m1: [-0.4375, 0.7175, 0.1032]<br>m2: [0.3535, 0.0950, 0.9180]	m0: [0.055518, 0.512697, 0.104886]<br>m1: [0.479319, 0.056323, 0.224097]<br>m2: [0.113827, 0.089921, 0.046088]
**Vertex**	m0: [0.5353, 0.5522, -0.0669]<br>m1: [-0.5539, 0.5281, -0.0418]<br>m2: [-0.0065, 0.0238, 0.9852]	m0: [0.527631, 0.344347, 0.084142]<br>m1: [0.354157, 0.521055, 0.124936]<br>m2: [0.052016, 0.159998, 0.021301]

Supplementary Table 1. Average coil orientation and variability. Values represent the average directional vector and standard deviations for the coil coordinate system.The vector m0 represents the transverse axis, m1 represents the handle direction, and m2 represents the coil normal. Low variances in m2 indicates that the coil was consistently placed tangentially against the scalp. Higher variance in m1 in DAN and Vertex targets indicates rotational adjustments to accommodate individual skull curvature.

Robustness Check: Controlling for Head Motion

To ensure that baseline head motion during the resting-state MRI scan did not drive the observed moderation effects, we ran an additional linear mixed-effects model including Mean Framewise Displacement (FD) as a covariate. The model structure was:

*v ~ L-FPN/M-FPN * Site * Stimulation + Days + FD + (1 | Participant)*

Results indicated that mean FD was not a significant predictor of the generalized drift rate ($t_{19.03}$ = -1.60; p = .13). Furthermore, the inclusion of this covariate did not meaningfully change our primary findings. The significant interaction between baseline connectivity, stimulation site (D-FPN relative to vertex), and timepoint remained

significant ($t_{93.14}$ = -2.24; p = .028) and in the same direction, confirming that our findings are robust to motion artifacts.

Robustness Check: Controlling for Global Signal Regression (GSR)

To ensure that GSR during fMRI preprocessing did not drive our main stimulation effects, we ran preprocessing again using aCompCor instead of GSR to extract our baseline network measures. All other methodologies were the same.

Our main contrasts showed the same direction of effects. Connectivity had a significant interaction for L-FPN-B vs. D-FPN stimulation (t_{104} = 2.26; p = .026). The connectivity interaction with L-FPN-B stimulation was positive relative to vertex (t_{104} = 1.14; p = .026), whereas the interaction for D-FPN stimulation relative to vertex was significantly negative (t_{104} = -1.13; p = .026). These findings indicate that the observed network moderation of TMS effects is not an artifact of the nuisance regression strategy, but rather related to individual differences in network architecture.

References

Andrews-Hanna, J. R., Smallwood, J., & Spreng, R. N. (2014). The default network and self-generated thought: Component processes, dynamic control, and clinical relevance. *Annals of the New York Academy of Sciences*, *1316*(1), 29–52. https://doi.org/10.1111/nyas.12360

Biswal, B., Yetkin, F. Z., Haughton, V. M., & Hyde, J. S. (1995). Functional connectivity in the motor cortex of resting human brain using echo-planar MRI. *Magnetic Resonance in Medicine*, *34*(4), 537–541. https://doi.org/10.1002/mrm.1910340409

Dale, A. M., Fischl, B., & Sereno, M. I. (1999). Cortical surface-based analysis. I. Segmentation and surface reconstruction. *NeuroImage*, *9*(2), 179–194. https://doi.org/10.1006/nimg.1998.0395

Dengler, J., Deck, B. L., Stoll, H., Fernandez-Nunez, G., Kelkar, A. S., Rich, R. R., Erickson, B. A., Erani, F., Faseyitan, O., Hamilton, R. H., & Medaglia, J. D. (2024). Enhancing cognitive control with transcranial magnetic stimulation in subject-specific

frontoparietal networks. *Cortex; a Journal Devoted to the Study of the Nervous System and Behavior*, *172*, 141–158. https://doi.org/10.1016/j.cortex.2023.11.020

Dixon, M. L., Andrews-Hanna, J. R., Spreng, R. N., Irving, Z. C., Mills, C., Girn, M., & Christoff, K. (2017). Interactions between the default network and dorsal attention network vary across default subsystems, time, and cognitive states. *NeuroImage*, *147*, 632–649. https://doi.org/10.1016/j.neuroimage.2016.12.073

Dixon, M. L., Vega, A. D. L., Mills, C., Andrews-Hanna, J., Spreng, R. N., Cole, M. W., & Christoff, K. (2018). Heterogeneity within the frontoparietal control network and its relationship to the default and dorsal attention networks. *Proceedings of the National Academy of Sciences*, *115*(7), E1598–E1607. https://doi.org/10.1073/pnas.1715766115

Esteban, O., Markiewicz, C. J., Blair, R. W., Moodie, C. A., Isik, A. I., Erramuzpe, A., Kent, J. D., Goncalves, M., DuPre, E., Snyder, M., Oya, H., Ghosh, S. S., Wright, J., Durnez, J., Poldrack, R. A., & Gorgolewski, K. J. (2019). fMRIPrep: A robust preprocessing pipeline for functional MRI. *Nature Methods*, *16*(1), Article 1. https://doi.org/10.1038/s41592-018-0235-4

Fonov, V., Evans, A. C., Botteron, K., Almli, C. R., McKinstry, R. C., & Collins, D. L. (2011). Unbiased Average Age-Appropriate Atlases for Pediatric Studies. *NeuroImage*, *54*(1), 313–327. https://doi.org/10.1016/j.neuroimage.2010.07.033

Gordon, E. M., Laumann, T. O., Gilmore, A. W., Newbold, D. J., Greene, D. J., Berg, J. J., Ortega, M., Hoyt-Drazen, C., Gratton, C., Sun, H., Hampton, J. M., Coalson, R. S., Nguyen, A. L., McDermott, K. B., Shimony, J. S., Snyder, A. Z., Schlaggar, B. L., Petersen, S. E., Nelson, S. M., & Dosenbach, N. U. F. (2017). Precision Functional Mapping of Individual Human Brains. *Neuron*, *95*(4), 791-807.e7. https://doi.org/10.1016/j.neuron.2017.07.011

Klein, A., Ghosh, S. S., Bao, F. S., Giard, J., Häme, Y., Stavsky, E., Lee, N., Rossa, B., Reuter, M., Chaibub Neto, E., & Keshavan, A. (2017). Mindboggling morphometry of

human brains. *PLoS Computational Biology*, *13*(2), e1005350. https://doi.org/10.1371/journal.pcbi.1005350

Li, M., Wang, D., Ren, J., Langs, G., Stoecklein, S., Brennan, B. P., Lu, J., Chen, H., & Liu, H. (2019). Performing group-level functional image analyses based on homologous functional regions mapped in individuals. *PLOS Biology*, *17*(3), e2007032. https://doi.org/10.1371/journal.pbio.2007032

Lowe, M. J., Dzemidzic, M., Lurito, J. T., Mathews, V. P., & Phillips, M. D. (2000). Correlations in Low-Frequency BOLD Fluctuations Reflect Cortico-Cortical Connections. *NeuroImage*, *12*(5), 582–587. https://doi.org/10.1006/nimg.2000.0654

Lynch, C. J., Power, J. D., Scult, M. A., Dubin, M., Gunning, F. M., & Liston, C. (2020). Rapid Precision Functional Mapping of Individuals Using Multi-Echo fMRI. *Cell Reports*, *33*(12), 108540. https://doi.org/10.1016/j.celrep.2020.108540

MacLeod, C. M. (2005). The Stroop Task in Cognitive Research. In *Cognitive methods and their application to clinical research* (pp. 17–40). American Psychological Association. https://doi.org/10.1037/10870-002

Navon, D. (1977). Forest before trees: The precedence of global features in visual perception. *Cognitive Psychology*, *9*(3), 353–383. https://doi.org/10.1016/0010-0285(77)90012-3

Reuter, M., Rosas, H. D., & Fischl, B. (2010). Highly accurate inverse consistent registration: A robust approach. *NeuroImage*, *53*(4), 1181–1196. https://doi.org/10.1016/j.neuroimage.2010.07.020

Ulrich, R., Schröter, H., Leuthold, H., & Birngruber, T. (2015). Automatic and controlled stimulus processing in conflict tasks: Superimposed diffusion processes and delta functions. *Cognitive Psychology*, *78*, 148–174. https://doi.org/10.1016/j.cogpsych.2015.02.005

Wang, D., Buckner, R. L., Fox, M. D., Holt, D. J., Holmes, A. J., Stoecklein, S., Langs, G., Pan, R., Qian, T., Li, K., Baker, J. T., Stufflebeam, S. M., Wang, K., Wang, X., Hong, B., & Liu, H. (2015). Parcellating cortical functional networks in individuals. *Nature Neuroscience*, *18*(12), Article 12. https://doi.org/10.1038/nn.4164
